# Effects of active vitamin D on insulin resistance and islet β-cell function in non-diabetic chronic kidney disease patients: a randomized controlled study

**DOI:** 10.1007/s11255-021-02968-7

**Published:** 2021-11-22

**Authors:** Yongxin Lu, Yi’an Wang, Yang Sun, Yongyan Li, Jingrui Wang, Yanhong Zhao, Fang Yang, Xiufang Gao, Jianqing Xu, Zongwu Tong

**Affiliations:** 1 Department of Nephrology, People’s Hospital of Yuxi City, The Sixth Affiliated Hospital of Kunming Medical University, Yuxi City, 653100 Yunnan Province China; 2 Department of Internal Medicine, Beicheng Health Center, Hongta District, Yuxi City, 653100 Yunnan Province China

**Keywords:** Active vitamin D, Non-diabetic chronic kidney disease, Insulin resistance, Islet β-cell function

## Abstract

**Purpose:**

The purpose of the study is to observe the effects of active vitamin D supplementation on insulin resistance and islet β-cell function (HOMA-β) in patients with non-diabetic chronic kidney disease (NDCKD).

**Methods:**

A total of 134 patients with NDCKD who met the inclusion criteria were enrolled in the prospective controlled study and categorized as such: 60 patients in the non-dialysis (ND) group; 36, hemodialysis (HD) group; and 38, peritoneal dialysis (PD) group. Each group was divided into two equal-numbered subgroups for vitamin D supplementation. Those in the experimental subgroups received calcitriol 0.5 ug/day orally, and were followed-up for 6 months. A total of 117 patients were followed-up, including 57 patients in the ND group; 29, HD group; and 31, PD group. Changes in the insulin resistance index (HOMA-IR) and HOMA-β index were calculated and compared at the time of enrollment and after 1, 3, and 6 months of intervention.

**Results:**

(1) Mean HOMA-IR value: In the ND group, mean HOMA-IR value of the experimental group significantly decreased compared with that of the control group after 3 months of intervention (*P* = 0.02). In the HD and PD groups, there was no statistical difference between the experimental and control groups (*P* > 0.05). (2) Mean HOMA-β index: In the ND group, mean HOMA-β index of the experimental group was higher than that of the control group after 1 month of active vitamin D treatment (*P* = 0.03), and, with an extended intervention time, the index gradually increased (*P* < 0.001). In the HD group, mean HOMA-β index of the experimental group was higher than that of the control group after 3 months of active vitamin D treatment (*P* = 0.01). Among PD patients, mean HOMA-β index of the patients in the experimental group was higher than that of the control group after 6 months of active vitamin D treatment (*P* = 0.02).

**Conclusions:**

Active vitamin D supplementation improved insulin resistance and HOMA-β after 6 months in ND patients, but only improved HOMA-β in the dialysis patients, with no significant effect on insulin resistance.

## Introduction

Activated vitamin D is an important steroid hormone that regulates calcium and phosphorus homeostasis, participates in bone metabolism, and plays a physiological role mainly in the form of 1,25(OH)_2_D and its analogs. In patients with chronic kidney disease (CKD), activated vitamin D deficiency is very common due to impaired renal structure and endocrine function [1]. In the human body, the kidney is the primary site of 1,25(OH)_2_D synthesis, and in the early stage of CKD, the normal structure and function of the renal tubulointerstitium are impaired, and the amount or activity of the 1α hydroxylase decreases, which reduces the conversion of 25(OH) D to 1,25(OH)_2_D, and the level of 1,25(OH)2D in serum decreases [2]. At the same time, with the progression of CKD, serum phosphorus and serum FGF23 levels increase significantly, both of them could further aggravate 1,25(OH)_2_D deficiency by inhibiting 1α hydroxylase activity to reduce 1,25(OH)_2_D synthesis [3, 4]. A large body of evidence suggests that this deficiency contributes to abnormal glucose metabolism and exacerbates insulin resistance (IR) in patients with non-diabetic chronic kidney disease (NDCKD) [5–8]. In this study, we intended to further investigate the effect of activated vitamin D treatment on glucose metabolism in patients with NDCKD by applying activated vitamin D analogs and observing the changes in the insulin resistance index (HOMA-IR) and pancreatic β-cell function (HOMA-β) of these patients after the use of this class of drugs.

## Materials and methods

### Study subjects

A total of 134 patients with NDCKD who visited the Department of Nephrology of the Sixth Affiliated Hospital of Kunming Medical University from March 2017 to March 2018 were selected for this prospective controlled study, including 60 patients in the non-dialysis (ND) group (male to female ratio 36:24, mean age 53.45 ± 17.32 years), 36 in the hemodialysis (HD) group (male to female ratio 18:18, mean age 48.75 ± 13.17 years), and 38 in the peritoneal dialysis (PD) group (male to female ratio 25:13, mean age 49.95 ± 13.69 years). Each treatment modality was randomly grouped equally, and a prospective controlled study was conducted. The study was approved by the ethical committee of the hospital, and the included subjects and their families were informed and signed the consent forms.

### Inclusion and exclusion criteria

The inclusion criteria are as follows: (1) meeting the diagnostic criteria of CKD: renal injury or estimated glomerular filtration rate < 60 mL/min/1.73 m^2^ for a period of at least 3 months. Renal injury was defined as: abnormal renal pathology or abnormal blood, urine, or imaging studies; (2) the patient has the ability to understand and communicate; (3) clinical stability; and (4) age > 18 years. The exclusion criteria are as follows: (1) patients with diabetes mellitus; (2) patients receiving glucocorticoids or other drugs affecting basal metabolic rate, such as for the treatment of abnormal thyroid function; (3) patients with acute renal insufficiency from various causes; (4) patients with acute infectious diseases; (5) patients with severe liver disease, active autoimmune diseases, and/or severe organ dysfunction; (6) patients with cognitive and communication impairment and/or mental instability; and (7) patients who refused to cooperate.

### Methods

Basic information on age, sex, height, weight, systolic and diastolic blood pressure, and related biochemical indices were collected at enrollment. Patients who met the enrollment criteria were randomly divided into experimental and control groups. Patients in the experimental group were given oral calcitriol (Rocaltrol, Roche Pharmaceuticals) 0.5 ug/day for 6 months after a 2-week lead-in period (discontinuation of vitamin D agent, natural vitamin D, and calcium), while those in the control group did not take the drug. The rest of the treatment was approximately the same as prior to the experiment. A total of 117 patients were followed-up, including 57 in the ND group, 29 in the HD group, and 31 in the PD group. The patients’ laboratory indices, such as urea nitrogen (Urease UV rate method), blood creatinine (sarcosine oxidase method), uric acid (uricase colorimetric method), total cholesterol (cholesterol oxidase method), triglycerides (GPO-POD(UV) enzymatic method), calcium ('5 nitro5 methyl bis-aminophenoxy ethylamine tetraacetic acid (NM-BAPTA) colorimetric method), iron (ferrozine colorimetric method), bicarbonate (enzymatic method), lactate (lactate oxidase method), fasting glucose (Hexokinase method), Hs-CRP (immunoturbidimetric method); Instruments: Roche cobas C 701 automatic biochemical analyzer imported from Switzerland; fasting serum insulin (chemiluminescent immunoassay method), 25(OH)D (include 25(OH) D_3_, 25(OH) D_2_ and 25(OH) D metabolites, chemiluminescent immunoassay method), and intact parathyroid hormone (iPTH, chemiluminescent immunoassay method); Instruments: Soling, Germany. All above indicators were determined at the time of enrollment, as well as after 1, 3, and 6 months of intervention, respectively. The main evaluation indices were as follows: (1) HOMA-IR and HOMA-β: the HOMA-IR and HOMA-β were calculated by applying the currently commonly used HOMA2 calculator 2.2.3 software [9], the expression of which is: HOMA-IR = fasting insulin (pmol/L) × fasting glucose (mmol/L)/22.5; (2) HOMA-β = 20 × fasting insulin (pmol/L)/(fasting glucose (mmol/L)—3.5).

### Statistical methods

SPSS 21 statistical software was applied, and *P* < 0.05 (two-sided) was considered statistically significant. The measurement data conforming to normal distribution were expressed as (± s), and the measurement data not conforming to normal distribution were expressed by rank-sum test. Comparisons of enumeration data and rates between two or more groups were performed using the chi-squared test.

## Results

### Baseline data

There were no statistical differences between the experimental and control groups in terms of age, sex ratio, body mass index, blood glucose, fasting insulin, calcium, phosphorus, Hs-CRP, and 25(OH)D levels at the time of enrollment in each group (see Table 1).

**Table 1:** Baseline information at entry for each group

Clinical indicator	Non-dialysis group			Hemodialysis group			Peritoneal dialysis group		
	Control group (*n* = 30)	Experimental group (*n* = 30)	Pa	Control group (*n* = 18)	Experimental group (*n* = 18)	Pb	Control group (*n* = 19)	Experimental group (*n* = 19)	Pc
Age (years)	56.07 ± 14.49	50.83 ± 19.64	0.11	47.5 ± 13.38	50.0 ± 13.21	0.95	45.11 ± 10.96	54.79 ± 14.68	0.23
Sex (m/f)	19/11	18/12	/	9/9	9/9	/	14/5	11/8	
BMI (kg/m2)	22.63 ± 3.84	22.98 ± 3.15	0.29	22.39 ± 3.29	22.73 ± 4.14	0.35	23.52 ± 3.88	24.03 ± 4.31	0.66
GLU mmol/L	4.92 ± 0.53	4.71 ± 0.61	0.46	4.81 ± 0.86	4.93 ± 0.84	0.93	4.83 ± 0.57	4.81 ± 0.58	0.90
TC mmol/L	4.64 ± 3.10	4.21 ± 1.38	0.09	3.99 ± 1.35	3.63 ± 1.09	0.40	5.15 ± 1.61	5.22 ± 1.60	0.97
TG mmol/L	1.93 ± 1.07	1.51 ± 0.73	0.16	1.75 ± 1.23	1.47 ± 0.52	0.26	1.95 ± 2.12	1.63 ± 1.62	0.27
Fasting insulin mmol/L	11.33 ± 8.62	9.17 ± 2.12	0.06	9.65 ± 10.39	8.89 ± 3.33	0.09	6.88 ± 2.07	6.84 ± 2.08	0.99
Ca mmol/L	2.09 ± 015	1.99 ± 0.24	0.08	2.01 ± 0.21	1.98 ± 0.37	0.07	2.01 ± 0.32	2.07 ± 0.23	0.16
P mmol/L	1.51 ± 0.49	1.56 ± 0.24	0.06	1.50 ± 0.38	1.54 ± 0.45	0.51	1.50 ± 0.37	1.53 ± 0.60	0.05
Hb (g/L)	111.2 ± 28.29	93.6 ± 25.21	0.54	91.5 ± 19.38	92.12 ± 23.95	0.39	108.95 ± 31.32	102.79 ± 22.17	0.15
Hs-CRP (mg/L)	13.1 ± 2.56	13.16 ± 3.36	0.15	13.40 ± 9.72	12.71 ± 7.91	0.55	11.81 ± 10.95	13.97 ± 7.18	0.39
25 (OH)D nmol/L	47.04 ± 14.56	44.76 ± 10.89	0.12	33.46 ± 13.69	35.91 ± 10.43	0.27	33.14 ± 6.98	36.54 ± 12.38	0.09

### Effects of activated vitamin D treatment on HOMA-IR in patients with NDCKD

**Fig. 1:**
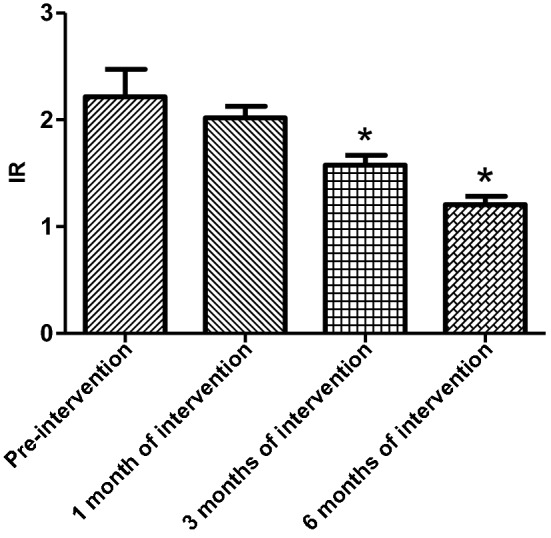
Change in insulin resistance index in the experimental group of non-dialysis patients (*P < 0.05)

**Fig. 2:**
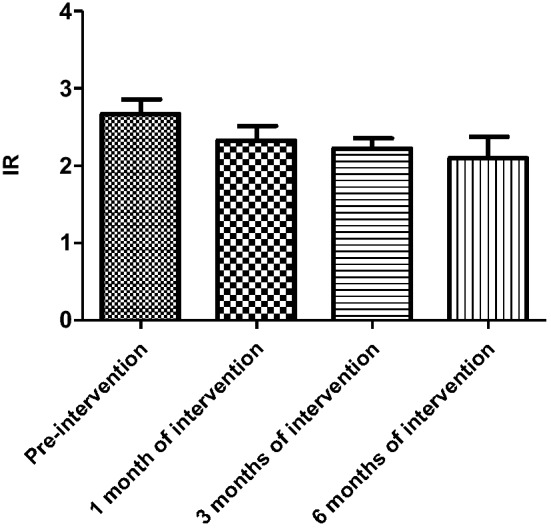
Change in insulin resistance index in the experimental group of hemodialysis patients

**Fig. 3:**
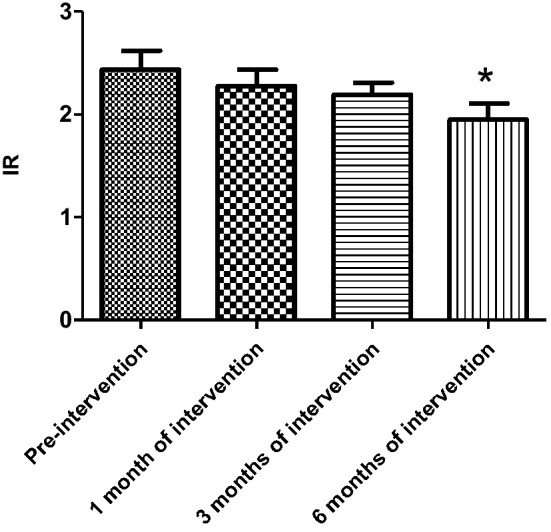
Change in insulin resistance index in the experimental group of peritoneal dialysis patients

HOMA-IR was not statistically different between the experimental and control subgroups in all three groups before the experimental intervention. The mean IR values of patients in the ND group started to decrease significantly after 3 months of intervention in the experimental group compared with the control group (3 months [March]: 2.31 ± 0.93 versus 1.58 ± 0.51, *P* = 0.02; 6 months [June]: 2.35 ± 0.88 versus 1.21 ± 0.43, *P* < 0.001). With the extension of the intervention time, compared with the beginning of the experiment, the IR values of patients in the experimental group also gradually decreased, and statistical difference was observed at 3 months of intervention (see Table 2 and Fig. 1). In the HD and PD groups, the mean IR values were lower in the experimental group than in the control group, but there was no statistical difference (*P* > 0.05). With the extension of the intervention time, the IR values of patients within the experimental group tended to decrease, but there was no statistical difference between the groups (*P* > 0.05) (see Tables 3 and 4, Figs. 2 and 3).

**Table 2:** Changes in insulin resistance indices in the experimental and control groups of non-dialysis patients

IR value	Non-dialysis group		*P*
	Control group (*n* = 29)	Experimental group (*n* = 28)	
Pre-intervention	2.31 ± 1.85	2.22 ± 1.41	0.53
1 month of intervention	2.53 ± 0.84	2.02 ± 0.59	0.15
3 months of intervention	2.31 ± 0.93	1.58 ± 0.51	0.02
6 months of intervention	2.35 ± 0.88	1.21 ± 0.43	< 0.001

*IR* insulin resistance

**Table 3:** Changes in insulin resistance index in the experimental and control groups of hemodialysis patients

IR value	Hemodialysis group		*P*
	Control group (*n* = 14)	Experimental group (*n* = 15)	
Pre-intervention	2.58 ± 1.06	2.67 ± 0.79	0.85
1 month of intervention	2.53 ± 0.81	2.33 ± 0.81	0.23
3 months of intervention	2.49 ± 1.08	2.23 ± 0.55	0.17
6 months of intervention	2.50 ± 0.88	2.10 ± 1.17	0.15

*IR* insulin resistance

**Table 4:** Changes in insulin resistance index in experimental and control groups of peritoneal dialysis patients

IR value	Peritoneal dialysis group		*P*
	Control group (*n* = 15)	Experimental group (*n* = 16)	
Pre-intervention	2.49 ± 1.07	2.44 ± 0.8	0.65
1 month of intervention	2.39 ± 1.18	2.28 ± 0.69	0.42
3 months of intervention	2.67 ± 1.10	2.19 ± 0.53	0.25
6 months of intervention	2.38 ± 1.02	1.95 ± 0.69	0.12

*IR* insulin resistance

### Effects of activated vitamin D treatment on HOMA-β in patients with non-diabetic CKD

**Fig. 4:**
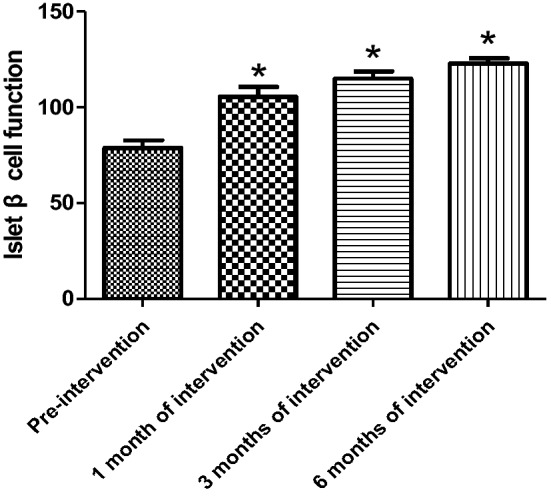
Change in HOMA-β index in the experimental group of non-dialysis patients (*P < 0.05)

**Fig. 5:**
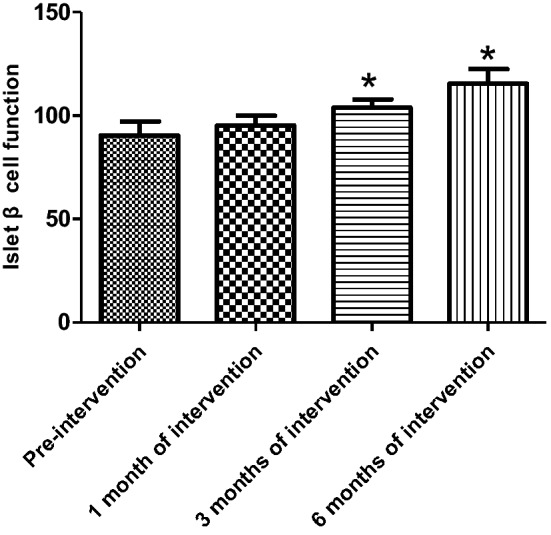
Change in HOMA-β index in the experimental group of hemodialysis patients (*P < 0.05)

**Fig. 6:**
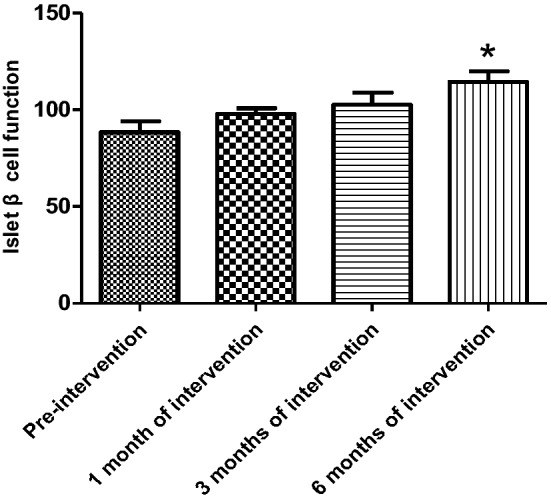
Change in HOMA-β index in the experimental group of peritoneal dialysis patients (*P < 0.05)

The HOMA-β index was not statistically different in all three groups before the experimental intervention compared with the control group. After 1 month of activated vitamin D treatment in ND patients, the HOMA-β index was elevated and statistically different in the experimental group compared with the control group (105.6 ± 27.59 versus 88.44 ± 21.92, *P* = 0.03) and gradually increased with longer intervention time, with statistical differences between control and experimental groups (3 months [March]): 89.96 ± 20.56 versus 115.0 ± 20.65, *P* < 0.001; 6 months: 86.10 ± 15.99 versus 123.0 ± 14.64, *P* < 0.001) (Table 5); and the improvement in the HOMA-β index was first observed to be statistically different in the experimental group after 1 month of intervention compared with that at the time of enrollment (Fig. 4). Among the HD patients, after 3 months of activated vitamin D treatment, the mean HOMA-β index was elevated in the experimental group compared with the control group, showing a statistical difference (3 months [March]: 89.96 ± 11.48 versus 103.80 ± 17.08, *P* = 0.01; 6 months [June]: 97.23 ± 17.44 versus 115.4 ± 30.14, *P* < 0.001) (Table 6). Among PD patients, the mean HOMA-β index was statistically higher in the experimental group compared with the control group after 6 months of activated vitamin D treatment (6 months [June]: 93.03 ± 18.68 versus 114.4 ± 22.5, *P* = 0.02) (Table 7). In the experimental intervention groups of HD and PD patients, as the duration of intervention increased, the patients' own control (compared with that at the time of enrollment) mean value of HOMA-β index tended to increase, with statistical differences in the experimental HD group at 3 months of intervention and in the PD group at 6 months of intervention compared with that at the time of enrollment, as shown in Figs. 5 and 6. In hemodialysis and peritoneal dialysis patients, we also observed changes in iPTH between the experimental group and the control group (Tables 8, 9), and the results showed that iPTH decreased significantly in both hemodialysis and peritoneal dialysis patients after treatment with active vitamin D.

**Table 5:** HOMA-β index in experimental and control groups among non-dialysis patients

	Non-dialysis group		*P*
	Control group (*n* = 29)	Experimental group (*n* = 28)	
Pre-intervention	86.40 ± 23.92	78.81 ± 21.91	0.25
1 month of intervention	88.44 ± 21.92	105.6 ± 27.59	0.03
3 months of intervention	89.96 ± 20.56	115.0 ± 20.65	< 0.001
6 months of intervention	86.10 ± 15.99	123.0 ± 14.64	< 0.001

**Table 6:** Changes in HOMA-β index in the experimental and control groups of hemodialysis patients

	Hemodialysis group		*P*
	Control group (*n* = 14)	Experimental group (*n* = 15)	
Pre-intervention	92.46 ± 16.93	90.26 ± 29.53	0.62
1 month of intervention	93.15 ± 13.43	95.17 ± 20.41	0.42
3 months of intervention	89.96 ± 11.48	103.80 ± 17.08	0.01
6 months of intervention	97.23 ± 17.44	115.4 ± 30.14	< 0.001

**Table 7:** Changes in HOMA-β index in the experimental and control groups of peritoneal dialysis patients

	Peritoneal dialysis group		*P*
	Control group (*n* = 15)	Experimental group (*n* = 16)	
Pre-intervention	88.33 ± 22.43	88.27 ± 24.57	0.88
1 month of intervention	94.03 ± 17.12	97.87 ± 13.03	0.62
3 months of intervention	97.21 ± 25.34	102.5 ± 28.03	0.12
6 months of intervention	93.03 ± 18.68	114.4 ± 22.5	0.02

**Table 8:** Changes of iPTH (pg/ml) in hemodialysis patients in experimental group and control group

	Hemodialysis group		*P*
	Control group (*n* = 14)	Experimental group (*n* = 15)	
Pre-intervention	571.73 ± 77.79	581.06 ± 83.61	0.75
1 month of intervention	581.11 ± 71.64	548.1 ± 63.47	0.15
3 months of intervention	592.62 ± 62.25	467.97 ± 109.80	0.0003
6 months of intervention	559.08 ± 49.64	441.92 ± 84.58	< 0.0001

**Table 9:** Changes of iPTH (pg/ml) in peritoneal dialysis patients in the experimental group and the control group

	Peritoneal dialysis group		*P*
	Control group (*n* = 15)	Experimental group (*n* = 16)	
Pre-intervention	485.90 ± 61.73	477.51 ± 63.35	0.68
1 month of intervention	490.97 ± 60.99	453.68 ± 30.12	0.11
3 months of intervention	496.82 ± 83.84	433.33 ± 146.78	0.02
6 months of intervention	509.06 ± 115.12	324.05 ± 74.61	< 0.0001

*Pa* experimental versus control *P* value for non-dialysis patients, *Pb* experimental versus control *P* value for hemodialysis patients, *Pc* experimental versus control *P* value for peritoneal dialysis patients, *BMI* body mass index

## Discussion

Current mechanisms of vitamin D regulation on glucose metabolism include: direct activation of vitamin D receptor or interference with vitamin D response elements in the insulin receptor promoter gene region affecting the function of pancreatic beta cells [10]; It can also improve insulin sensitivity and glucose transport by enhancing insulin receptor response to insulin [11]. Recent studies have shown that active vitamin D may also play a role in reducing food intake, reducing body weight, improving glucose tolerance, and insulin sensitivity through the hypothalamic paraventricular nucleus vitamin D receptor [12]. Studies suggest that IR is a common and early alteration in patients with CKD [13], especially in dialysis patients where it is prevalent [14, 15], and that dialysis has an effect on IR. HD patients with IR can have reduced serum insulin concentrations after dialysis, resulting in a relative deficiency of blood insulin, as well as post-dialysis hyperglycemia due to increased hepatic gluconeogenesis capacity following HD removal of toxins [16]. In the case of PD, because the PD fluid contains glucose, a persistent high glucose load is considered a risk factor for IR and disorders of glucose metabolism in the body, and studies have found that insulin sensitivity is significantly lower in PD patients with non-diabetic kidney disease than in HD patients [17].

A large number of studies have confirmed that the factors affecting IR in patients with CKD are complex and diverse, mainly including active vitamin D deficiency, inflammation, oxidative stress, obesity, metabolic acidosis, anemia, and microbial toxins. These factors lead to IR by affecting the body's glucose uptake and utilization. Among the factors affecting IR, vitamin D levels have become a hot spot for research in recent years because it not only regulates calcium and phosphorus metabolism, but also affects several systems of the body, including neurological, endocrine, immune, and reproductive systems. Basic research suggests that vitamin D may possess antioxidant properties by modifying some of the antioxidant enzymes, thus protecting pancreatic β-cells against apoptosis [18]. A recent clinical study also suggested that the prevalence of metabolic syndrome and diabetes is significantly lower in individuals with serum 25(OH)D levels > 75 nmol/L [19]. In patients with NDCKD, there is a general decrease in serum 25(OH)D levels due to decreased renal function, reduced intake, inadequate sun exposure, and toxins, which leads to islet cell calcium channel closure, blocked insulin receptor substrate phosphorylation, and impaired glucose uptake, thus resulting in IR [20]. In addition, 25(OH)D deficiency can cause secondary hyperparathyroidism (SHPT), and high parathyroid hormone (PTH) levels can inhibit insulin secretion, further aggravating IR [21]. One study found that supplementation with active vitamin D improved IR in patients with NDCKD [22]. However, there are also additional findings suggesting that active vitamin D supplementation in humans improves insulin sensitivity only in patients with early diabetes, but not insulin sensitivity and β-cell function in other patients [23]. It was found in the preliminary epidemiological investigation of this study: the incidence of IR in patients with CKD with 25(OH)D deficiency (< 50 nmol/L) (44%) was also found to be significantly higher than in the 25(OH)D normal group (> 50 nmol/L) (21%) during the pre-enrollment screening of patients, and the difference was statistically significant (*P* < 0.05), suggesting that 25(OH)D deficiency is associated with the development of IR in patients with CKD, which was in line with previous clinical research [24]. However, although several observational studies and meta-analyses have shown a positive association between circulating 25(OH)D_3_ concentration and the risk of type 2 diabetes, no randomized clinical trial of active vitamin D supplementation has ultimately provided sufficient evidence to confirm this hypothesis [25]. Therefore, we designed such a preliminary intervention study in patients with non-diabetic CKD and obtained some preliminary results.

In this study, after therapeutic intervention with active vitamin D (1, 25(OH)_2_D_3_, calcitriol) supplementation in patients with NDCKD under different treatment modalities, there was a statistically significant improvement in IR and islet β-cell function after active vitamin D supplementation in the experimental ND group compared with the control group, and this result can be verified with the results of a previous clinical correlation study [26]. However, there was no significant improvement in IR index after 6 months of follow-up with active vitamin D supplementation in the experimental dialysis patients compared with the control group. However, the patients in the experimental group showed an overall decreasing trend in IR index with the prolongation of the intervention time, and all showed statistical differences at 6 months of follow-up; the observation of HOMA-β index showed a significant improvement in HOMA-β with the prolongation of the experimental time in both HD and PD patients, both compared with the control group and to the experimental group itself. This may suggest that because the degree of IR in dialysis patients is severe, longer clinical interventions may be needed to improve the function of pancreatic β-cells and thus the IR status of patients; or that there are other confounding factors affecting glucose metabolism in dialysis patients. These implications should be verified by further in-depth studies.

This study was based on previous studies wherein patients with NDCKD underwent activated vitamin D intervention for a relatively long period of time, and the relevant indices were observed for a similarly long period of time. The experimental conclusion of a positive effect on the improvement of insulin function was obtained from these studies, which can be used as a clinical reference basis for subsequent related studies. However, our study was just a pilot study, the shortcomings of this study include the data of a single-center study, the small number of experimental cases, the experimental process being unable to meet the standards of double-blind and placebo-controlled research, and the short follow-up time; Therefore, the experimental results are only for clinical treatment reference, subsequent research should involve a clinical randomized controlled trial with a larger scale, more rigorous design, and a longer follow-up period to further observe the effect of activated vitamin D on glucose metabolism in patients with non-diabetic kidney disease.
